# Growth behaviour and mechanical properties of PLL/HA multilayer films studied by AFM

**DOI:** 10.3762/bjnano.3.87

**Published:** 2012-11-21

**Authors:** Cagri Üzüm, Johannes Hellwig, Narayanan Madaboosi, Dmitry Volodkin, Regine von Klitzing

**Affiliations:** 1 Stranski-Laboratorium, Department of Chemistry, TU Berlin, Strasse des 17. Juni 124, D-10623 Berlin, Germany; 2 Fraunhofer Institute for Biomedical Engineering, Am Mühlenberg 13, 14476 Potsdam-Golm, Germany; 3 Max Planck Institute of Colloids and Interfaces, Am Mühlenberg 1, 14476 Potsdam-Golm, Germany

**Keywords:** atomic force microscopy, polyelectrolyte multilayers, stress relaxation, viscoelasticity, Young’s modulus

## Abstract

Scanning- and colloidal-probe atomic force microscopy were used to study the mechanical properties of poly(L-lysine)/hyaluronan (PLL/HA)*_n_* films as a function of indentation velocity and the number of polymer deposition steps *n*. The film thickness was determined by two independent AFM-based methods: scratch-and-scan and newly developed full-indentation. The advantages and disadvantages of both methods are highlighted, and error minimization techniques in elasticity measurements are addressed. It was found that the film thickness increases linearly with the bilayer number *n*, ranging between 400 and 7500 nm for *n* = 12 and 96, respectively. The apparent Young’s modulus *E* ranges between 15 and 40 kPa and does not depend on the indenter size or the film bilayer number *n*. Stress relaxation measurements show that PLL/HA films have a viscoelastic behaviour, regardless of their thickness. If indentation is performed several times at the same lateral position on the film, a viscous/plastic deformation takes place.

## Introduction

Polyelectrolyte multilayers (PEMs) have been studied intensely for the past two decades [1–2]. Despite their complex structure and wide range of applicability, PEMs can be prepared simply by alternating deposition of polycations and polyanions by dipping/spraying a substrate into/with the corresponding polyelectrolyte solutions. Potential applications of PEMs (e.g., filtration, paper making and biomaterials) require control of their adhesive behaviour by tuning the elastic/viscoelastic properties [3–5]. For example, the adsorption behaviour of proteins and cells, which is highly sensitive to the elasticity of the substrate, must be known and controlled for the development of contact lenses and antifouling materials [3,5–6]. Nevertheless, thin films have to be studied in the form in which they are available, making the use of macroscopic methods unsuitable. Therefore, scanning- or colloidal-probe atomic force microscopy have been widely used for studying the topography and the mechanical properties of PEMs [3–5,7–8].

One of the first measurements of elastic modulus with atomic force microscopy (AFM) on biological films was performed on lung-cancer cells, back in 1993 [9]. Further measurements include different strains of *E. coli* with a colloidal probe [10], elastic modulus of human platelet cells [11], human bone cell or skeletal muscle cells [12], breast cancer cells [13–14], hydrogel films [15–17], or nanoribbons [18], as well as single hydrogel particles [19–22]. Recent advances in the area have been summarized by Picart and co-workers [23–24].

Several studies on the bio-applicability of polymer-based films showed that if cells are deposited on a surface with an elasticity gradient, they move from the softer region to a relatively harder one [3,6,25]. Richert et al. showed that chondrosarcoma cells adhere much more strongly on chemically cross-linked poly(L-lysine)/hyaluronic acid PLL/HA films than on native PLL/HA films due to the elasticity difference between the two structures, the cross-linked surface being harder [6]. Engler et al. reported a similar spreading behaviour for smooth-muscle cells [3]. An enhanced cell growth was observed also for cross-linked chitosan/hyaluronan multilayer films, as compared to the native ones [8]. These reports suggest that polyelectrolyte multilayer films are ideal matrices for bio-applications as their elasticity can be tuned in a wide range simply by changing the cross-linker content in the structure. In that manner, PLL/HA films gained more attention than their analogues, and a wide range of Young’s moduli between 3 and 400 kPa was accessed by cross-linking (mostly with 1-ethyl-3-(3-(dimethylamino)propyl)carbodiimide, EDC) [3–5,7].

Although native (non-cross-linked) PLL/HA films were previously produced and well characterised, it is still a requirement to precisely measure not only the elasticity but also other rheological properties of these matrices on time scales suitable for biological processes [3]. In this work, scanning- and colloidal-probe AFM were used to perform nanoindentation on poly (L-lysine)/hyaluronan (PLL/HA)*_n_* films with *n* = 12–96, in order to better understand their growth behaviour, apparent Young’s modulus, and viscoelastic properties.

## Results and Discussion

### Bilayer number *n* versus film thickness *h*

**Figure 1:**
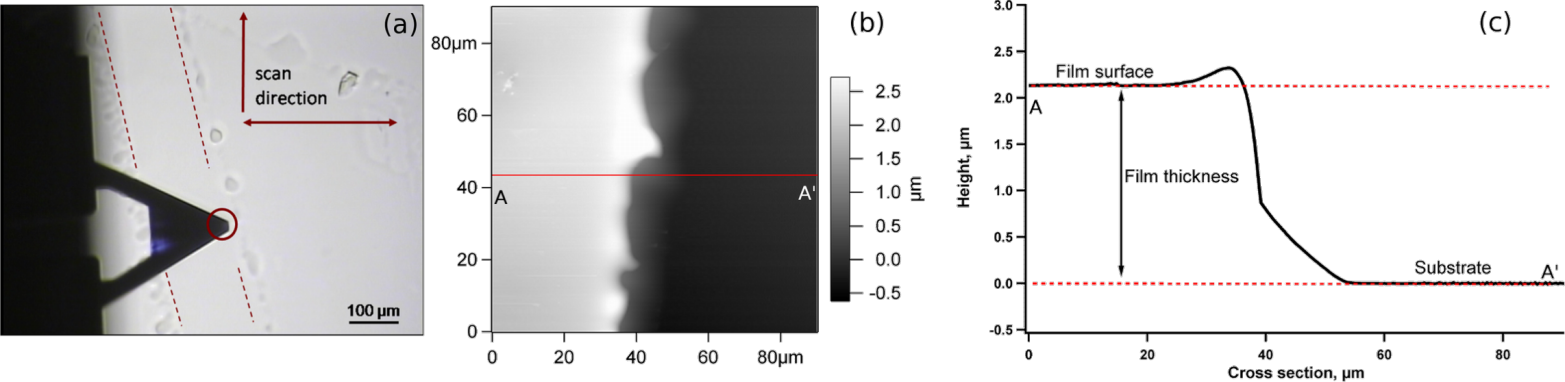
(a) Top camera view of the cantilever and the scratch on the film (delimited with the dashed lines). The circle points out the position of the tip. (b) 2-D AFM image of the scratch and the cross-section line (A–A′). The grey scale bar shows the height. (c) Height profile through the cross-section line (A–A′) for a measurement made on (PLL/HA)_36_.

The thickness *h* of (PLL/HA)*_n_* films with *n* = 12, 24, 36, 48, 60, 72, 84, 96 was measured both to determine the growth regime and to be able to study the mechanical properties. Two methods were used to determine the thickness. The first one is the scratch-and-scan method and comprises the removal of a small part of the film and subsequent imaging of the surface with a scanning-probe AFM. An optical microscope image of the scratched area on a PLL/HA film is shown in Figure 1 together with its AFM micrograph. The scratched area can be clearly seen due to its smoothness and lower height. In order to ensure that the film removal was successful, force measurements were made on this area and no indentation was observed. The thickness was extracted from the cross-section profile by calculating the height difference of the higher and the lower areas (excluding the rim) as shown in Figure 1c.

The scratch-and-scan method has some advantages such as the possibility to observe any possible damage done to the film during indentation, by scanning a large area that includes the originally studied position on the film. Another advantage is that this method gives the prerequisite image as well as the sample thickness in force-mapping studies [13–14,21–22,26–27]. The disadvantages mainly lie in the difficulty of obtaining a detailed image on soft surfaces, especially in liquid media. A magnetically driven cantilever as used in this study enhances the image quality since the surrounding medium is not excited to vibrations. On the other hand, this type of cantilever driving is still not available in most commercial systems and the appropriate cantilevers are still not common [28].

**Figure 2:**
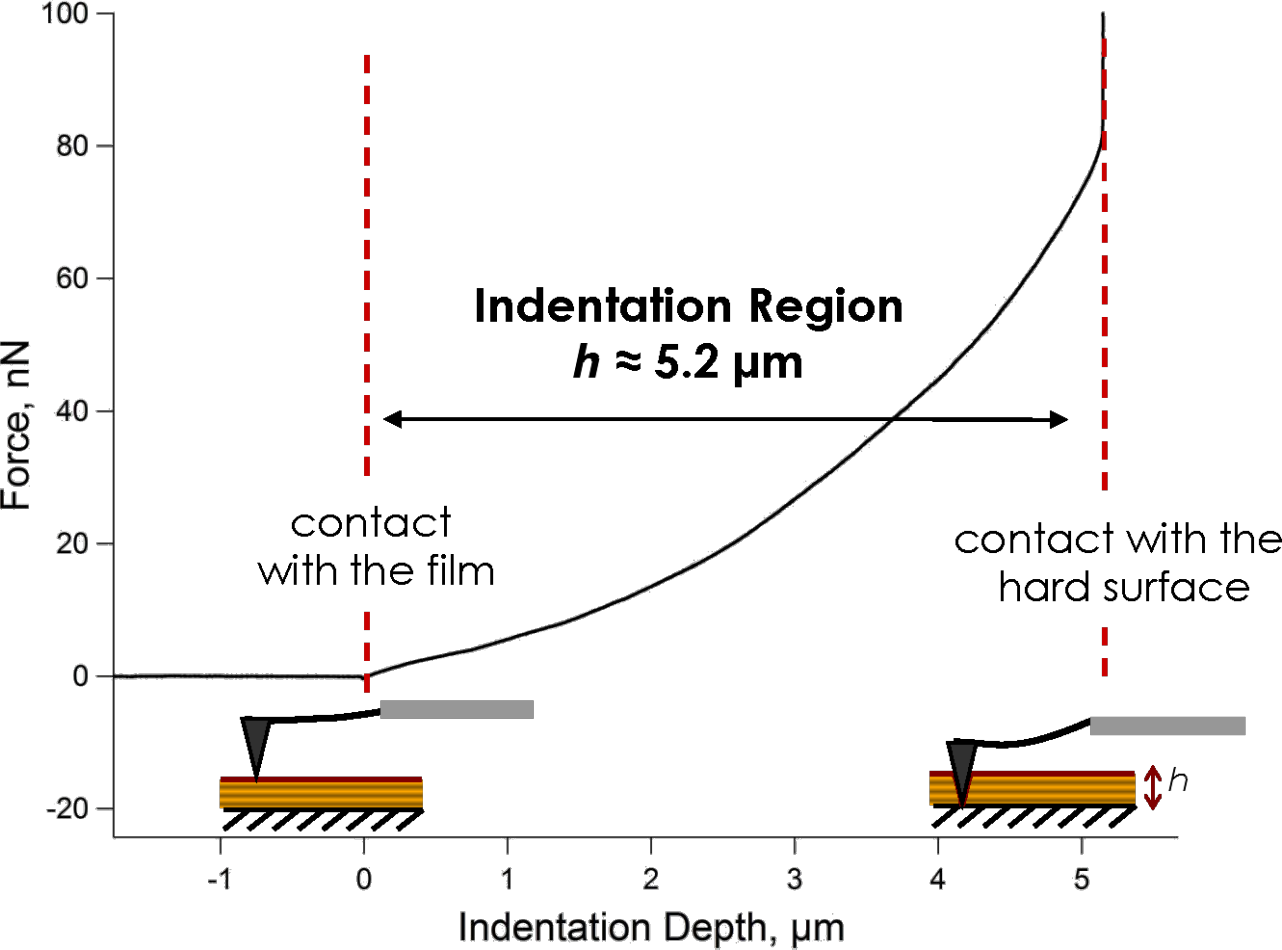
Calculation of the film thickness by the full-indentation method. The dashed lines show the tip-film and tip-substrate contact points. The measurement was made on (PLL/HA)_72_.

The second method to determine the film thickness is the full-indentation method, introduced in the experimental section. The total penetration depth in a force measurement has already been used to determine the thickness of nanometre-scale coatings, e.g., lipid bilayers [29], but, to our knowledge, the total thickness of micron-scale polymeric films has not yet been extracted in this way. The full-indentation method requires a stiff, calibrated cantilever equipped with a tip that is significantly longer than the film is thick. The measurements are reasonably fast and reproducible, and the film damage caused is limited to a small area (*r* ≈ 50 nm). The spring constant of the cantilever or the exact shape and size of the indenter do not have to be determined; however, an optical sensitivity calibration is required. A typical *F* versus *δ* curve for (PLL/HA)_72_ and determination of its thickness are presented in Figure 2.

**Figure 3:**
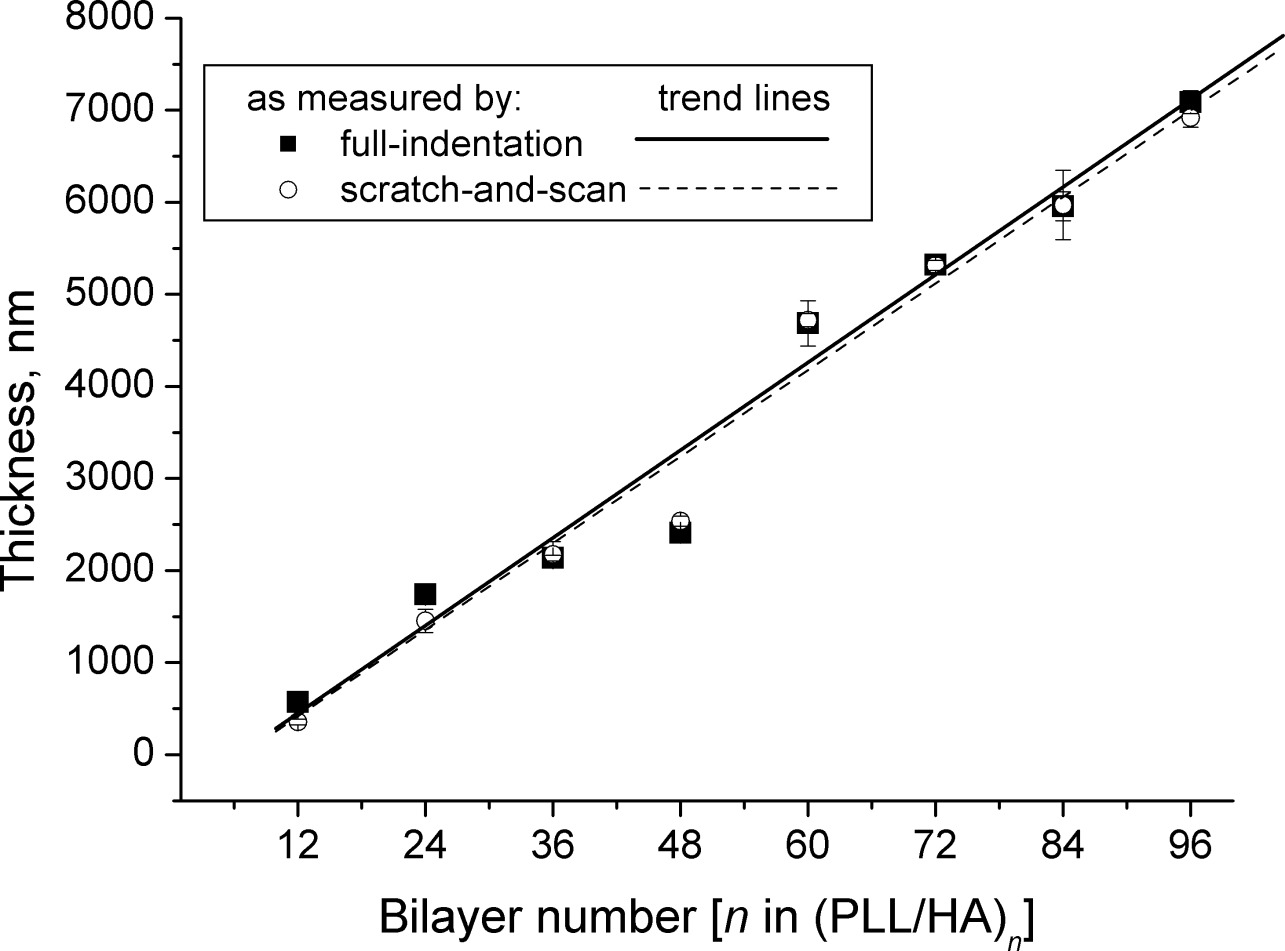
Thickness of (PLL/HA)*_n_* as a function of *n* as measured by scratch-and-scan and full-indentation methods. There is a good agreement between the two methods, and the thickness grows linearly with *n*. Error bars indicate the standard deviations.

A comparison of the film thickness obtained by scratch-and-scan and full-indentation is presented in Figure 3. The results of the two completely independent methods coincide very well within the experimental errors. Film thickness increases linearly with increasing bilayer number *n* (with an exception of *n* = 48) and ranges from about 0.4 to 7 μm for *n* = 12 and *n* = 96, respectively. Linear thickness growth with bilayer number indicates stacked polyelectrolyte layers that interpenetrate only into the neighbouring layers, forming periodic structures [5,24]. Although PLL/HA films are known to be highly hydrated and less ordered, it has been previously suggested that they grow exponentially [24,30–31] up to a certain bilayer number and thereafter the growth regime switches to a linear one [4,6,24,32–33]. For the dry state, a transition from exponential to linear growth was observed for *n* = 12–18 depending on the polyelectrolyte molecular weight [34–35]. Hence, our observation of a linear growth coincides well with the previous reports [4,6,24,32–33].

### The contact point and film thickness issues

**Figure 4:**
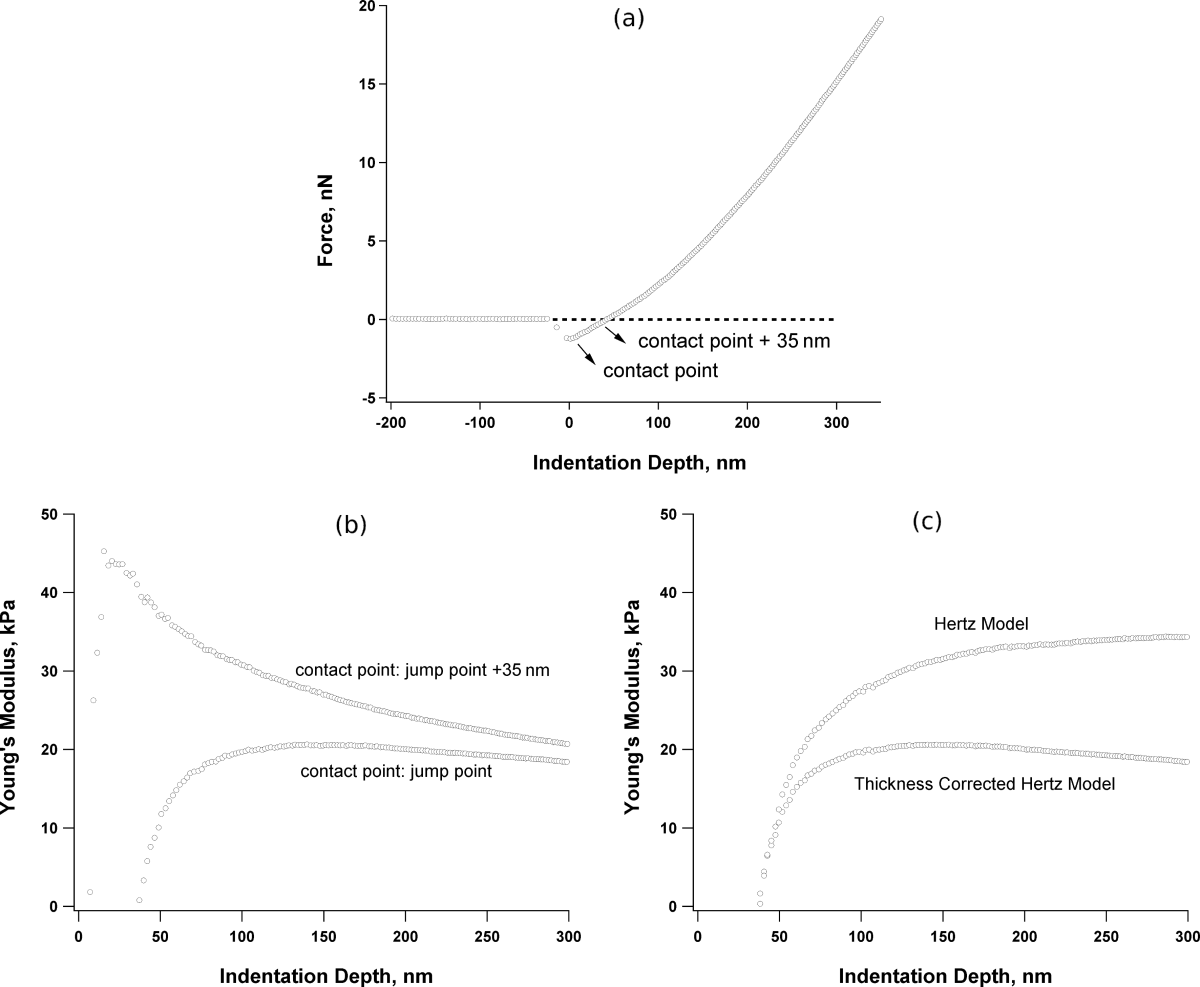
(a) A typical force–indentation depth curve as measured by CP-AFM on (PLL/HA)_24_. The jump point of the colloidal-probe to the film surface was taken as the contact point. (b) Young’s modulus versus indentation depth for two contact-point choices and (c) Young’s modulus calculated with the classical [36] and thickness-corrected [37] Hertzian models.

The Hertzian model [36] calculates the Young’s modulus *E* for each exact indentation depth, and thus a wrong indentation-depth determination may give rise to dramatic errors [37]. This problem is illustrated in Figure 4b.

Figure 4b shows the calculated Young’s modulus *E* versus the indentation depth *δ* for (PLL/HA)_24_ with two different contact points chosen on the raw *F*–*δ* curve as pointed out in Figure 4a. The first contact point is where the probe “jumps” to the surface, which can be recognized by an abrupt decrease in the measured force. The second contact point is chosen randomly at a further 35 nm, which is a clear shift considering the small film thickness. Although at deeper indentation depths the difference in the calculated *E* can be ignored, the deviation is dramatic in the region of small indentation depth, as previously reported [37–39]. In this study, the position of the minimum force was taken as the contact point, but in the case that there is no such minimum in the force curve, more effort is needed to determine an approximate contact point [21,37–38,40].

Another source of error in mechanical measurements of thin films is the substrate effect [37]. Although film stiffening due to the hard substrate is a real effect, it should be eliminated in the calculations since it does not reflect the material properties of the coating and may not be detected by the practical sensors, e.g., cells. Figure 4c shows the difference between *E* calculated by the classical Hertzian model [36] and the thickness-corrected one with Equation 1 [37], for (PLL/HA)_24_ (thickness ≈ 1500 nm). *E* calculated with the classical Hertzian model is nearly two fold larger than that from the thickness-corrected one. It was observed that the substrate effect is less stressed for thicker films, but it cannot be neglected for a film thinner than ≈5000 nm.

### Repetitive indentation measurements at one lateral position

**Figure 5:**
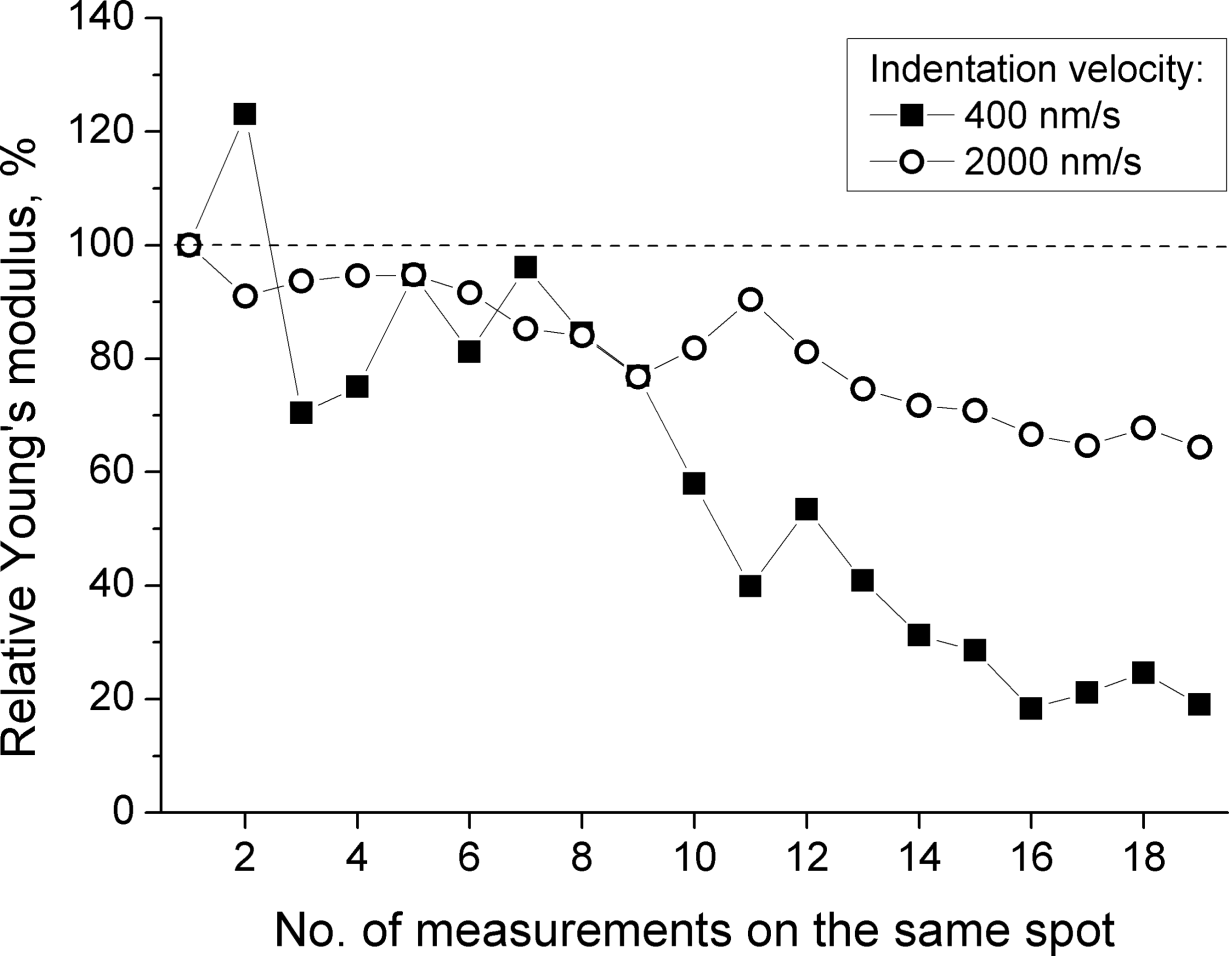
Relative percentage Young’s modulus *E*% for (PLL/HA)_72_ as measured on one fixed spot. All the values are normalised to the *E* calculated from the first indentation. Indentation velocities are 400 nm/s (squares) and 2000 nm/s (circles).

In order to test the reversibility of film deformation in the indentation studies, a series of force measurements were performed repetitively at one fixed lateral position. Two different indentation velocities, i.e., 400 and 2000 nm/s were used, each at a different lateral position. Enough time was left between any two sequential measurements to allow a complete relaxation of the film. *E* of (PLL/HA)_72_ as a function of the number of repeat measurements is shown in Figure 5 (*E* relative to the first measured one).

For both indentation velocities, repetitive measurements result in a continuous decrease in the apparent *E*, with the exception of some strong fluctuations, which are most likely measurement errors. The decrease is more stressed for slower indentation: *E* decreases by nearly 80% at 400 nm/s and by nearly 30% at 2000 nm/s. Presumably, the film is damaged once the applied load exceeds a certain limit, regardless of the indentation velocity, and it undergoes an irreversible viscous [41] or plastic deformation [42], leading to a thinner film and a softer structure. In order to avoid any irreversible deformation effects, in the following sections the lateral position of the measurement was changed each time after an indentation process.

### Indentation velocity versus Young’s modulus *E*

**Figure 6:**
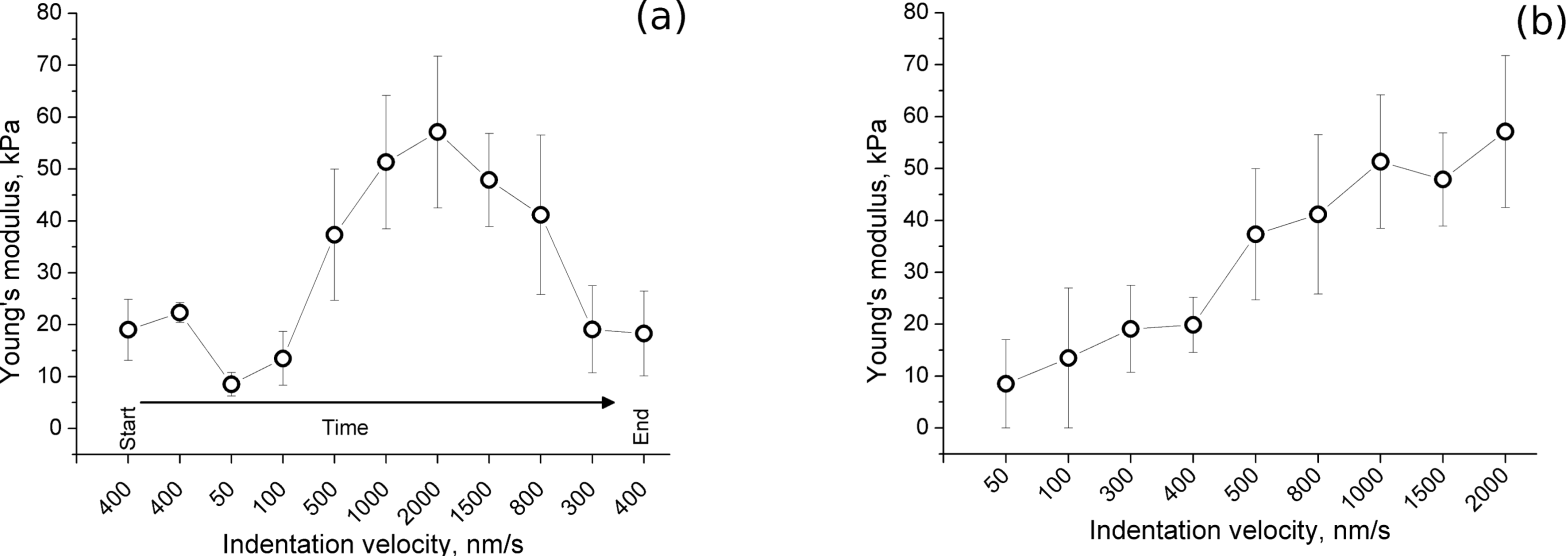
Calculated Young’s modulus *E* for (PLL/HA)_72_ with different indentation velocities sorted by (a) the measurement order (b) indentation velocity. Error bars show the standard deviation from 15 measurements.

Since the measurements take sometimes up to an hour, it was tested whether the mechanical properties of the film varied with time due to, e.g., instrument-based heating or contamination. The indentation velocity in the successive measurement sets was changed randomly in the following order: 400 nm/s, 400 nm/s, 50 nm/s, 100 nm/s, 500 nm/s, 1000 nm/s, 2000 nm/s, 1500 nm/s, 800 nm/s, 300 nm/s, 400 nm/s. The measurements were performed in a row without changing any further parameters except for the lateral position on the film. The dependence of the apparent *E* on the indentation velocity is shown in Figure 6a for (PLL/HA)_72_. In this graph, the indentation velocity values in the *x*-axis are sorted according to the order in which they were applied. The first two measurement sets with 400 nm/s gave the same *E* value as the other 400 nm/s set, which was made nearly two hours later, indicating that the measurement duration does not affect *E*, at least within a period of several hours.

On the other hand, *E* clearly depends on the indentation velocity. Figure 6b shows the same data sorted according to indentation velocity. The Young’s modulus *E* increases continuously from ≈10 kPa for 50 nm/s up to ≈60 kPa for 2000 nm/s. Previously, Francius et al. also showed that *E* of PLL/HA films was influenced by the indentation velocity [5]. They reported a nearly constant *E* below 500 nm/s, except for some fluctuations, and above that an increasing *E* with increasing indentation velocity. In the current study, *E* increases continuously even when the indentation velocity is below 500 nm/s. The difference between the two studies can be explained by differences in the nature of the films (due to the molecular weight of the polymers, preparation conditions, etc.) as well as in the measurement and data-handling procedures.

The dependence of *E* on the indentation velocity clearly indicates a viscoelastic film character [23,41] as will be further discussed below in terms of stress-relaxation measurements. In fact, due to the viscoelastic liquid character of the PLL/HA films, their equilibrium *E* is expected to be very low if not zero [23]. The Young’s modulus reported in this study refers to the *apparent* Young’s modulus, which arises as a reaction to a sudden load. Despite this fact, determining an apparent *E* on the time scale of cellular processes (<500 nm/s) gives a first insight into the film’s mechanical properties and makes a comparison of different surfaces possible [23]. Therefore, a fixed indentation velocity of 400 nm/s was used for the elasticity measurements in the following section.

### Effect of film thickness and indenter size on Young’s modulus

**Figure 7:**
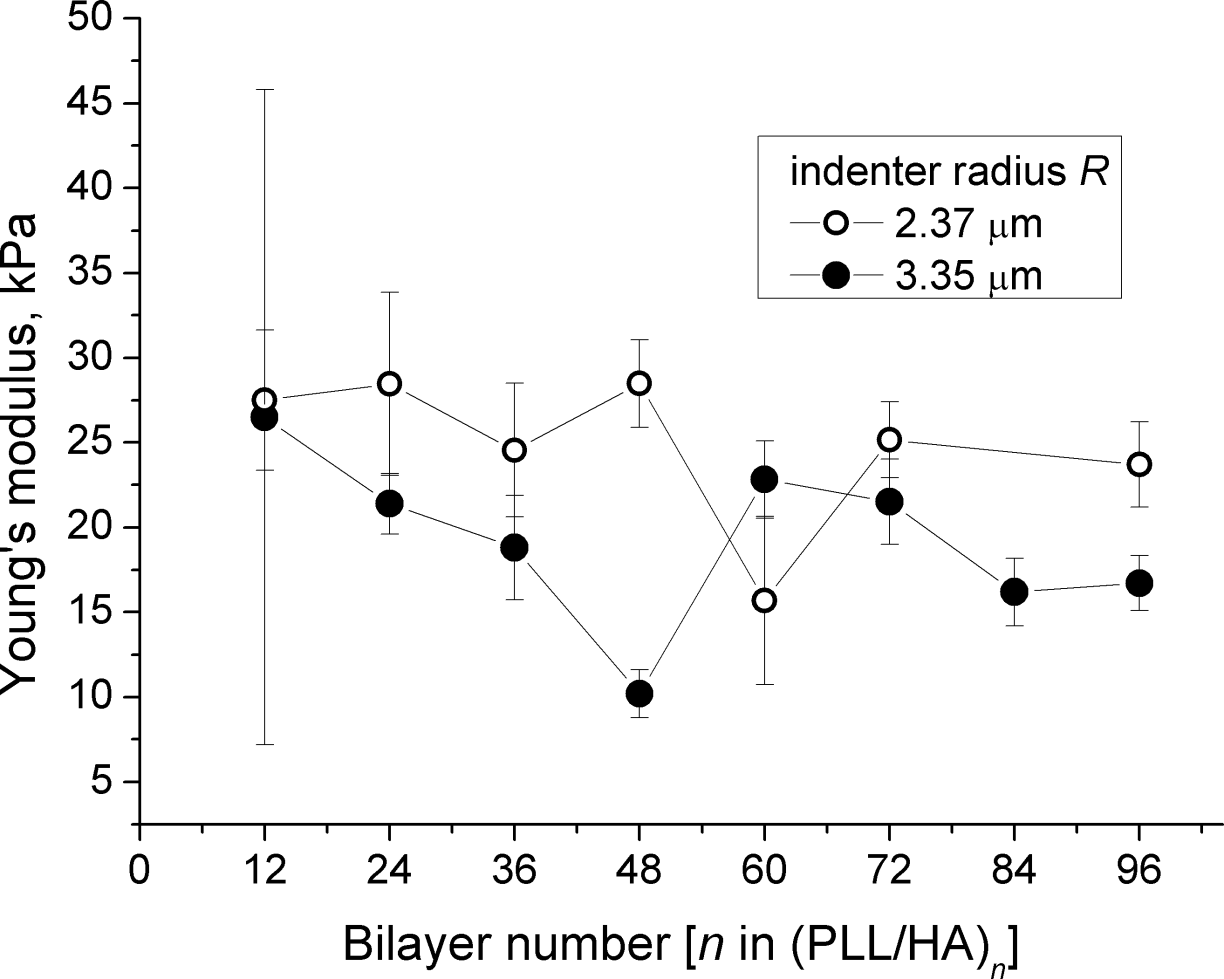
Young’s modulus *E* as a function of (PLL/HA)*_n_* bilayer number *n* for an indenter radius of 2.37 μm (open circles) and 3.35 μm (closed circles). Neither *n* nor *R* seem to affect the calculated *E* in a systematic way at an indentation velocity of 400 nm/s.

Small indenters, e.g., AFM tips, prevent errors due to insufficient indentation forces [15] and allow all scanning and force measurements to be done without changing any parameters [13,17,21]. On the other hand, shape and size determination of AFM tips is not straightforward, tips are more vulnerable to deformation during the measurements, and they can apply high loads on the film, invalidating the Hertzian assumptions [4,37,43]. A larger indenter, on the other hand, is advantageous due to the ease of size determination and attachment to the cantilever. In this study, tips were used for determination of the film thickness while colloidal probes were preferred in mechanical measurements. One way or another, the size of the indenter should not change the calculated Young’s modulus *E* since the indenter radius *R* is already included in Equation 1. Two colloidal probes that are 2.37 and 3.35 μm in radius *R* were compared in order to ensure the applicability of Equation 1 for the studied systems. The apparent *E* as a function of film bilayer number *n* and probe radius *R* is presented in Figure 7 showing indeed no systematic dependence of *E* on the indenter radius *R*.

Figure 7 suggests also that *E* does not change in a systematic way with the film bilayer number *n*. This result contradicts the results of Richert et al. [4], who reported a decrease in *E* from 90 kPa for *n* = 20 to 40 kPa for *n* = 60. The authors suggested a possibility of film softening due to greater hydration of the upper layers rather than due to a change in surface structure, heterogeneity or roughness. The film thickness range reported in the mentioned study [4] is nearly threefold larger than ours, which may result in significant structural differences.

Despite some fluctuations, *E* in our study ranges between 10 and 40 kPa, being in the same order of magnitude with the previous reports [3–5,7,44–45]. Only the film with 12 bilayers shows a very high variation in Young’s modulus, from one measurement position to the other. This can be explained by inhomogeneities on the film surface, which will affect the local film thickness dramatically compared to the thicker films. The determination of *E* is very sensitive to the thickness *h* for thinner films, as Equation 1 suggests: For larger *h* values, the correction terms on the right side approach 1, making Equation 1 independent of *h*. Another problem with thinner films is the difficulty of extracting the *F* versus *δ* data for the indentation depth at 5–10% of the total thickness. For very thin films, this range was extended up to 20% of the total thickness, restricted to cases for which the calculated *E* does not change abruptly.

### Relaxation time measurements

**Figure 8:**
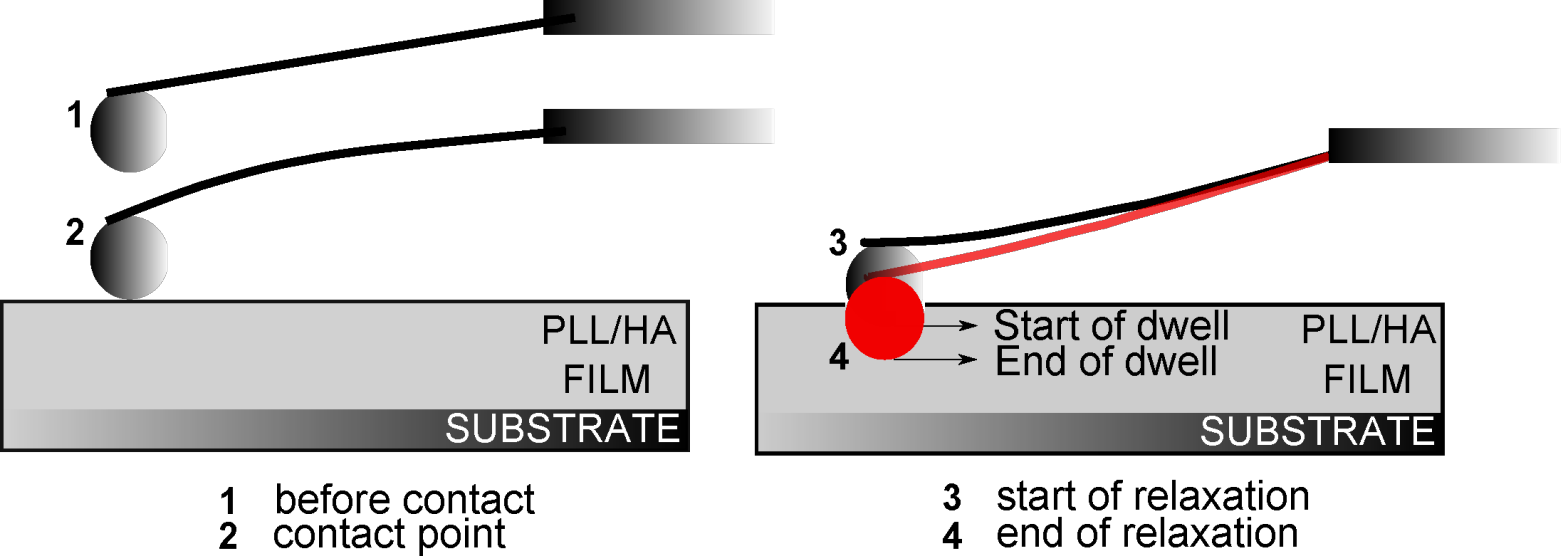
A summary of the stress-relaxation measurements. “Start of dwell” is the indentation depth at which the driving of the *z*-piezo is stopped. “End of dwell” shows the maximum indentation depth caused by the relaxation on the cantilever.

**Figure 9:**
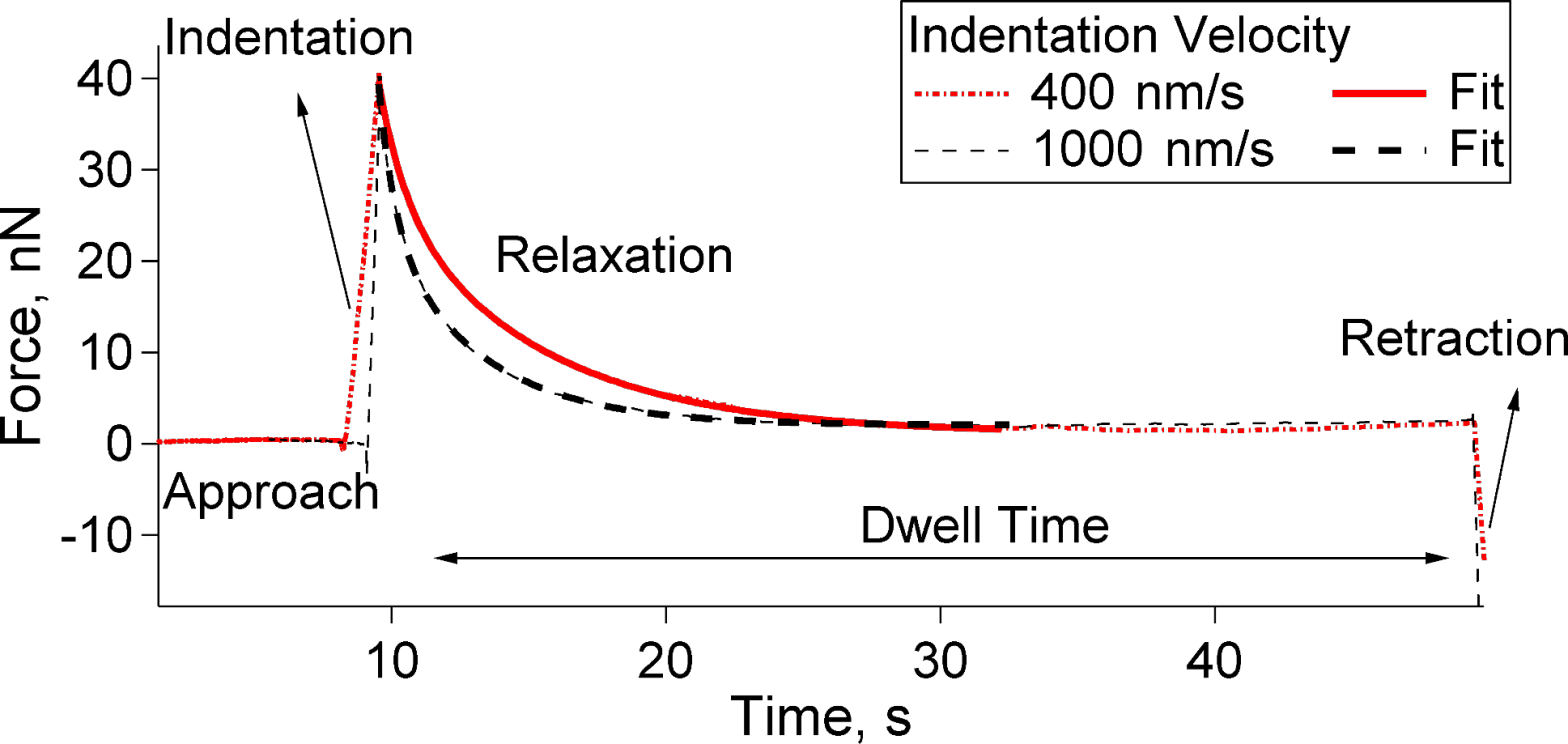
Stress relaxation curve with a dwell time of 40 s. The biexponential fit is represented by the thick lines on the decaying part of the force curve, in the dwell regime.

Measuring the viscoelastic properties of films in the thin-film limit can be challenging due to the requirement of precise information on the surface charge as well as on the film thickness, indentation depth and the indenter shape/size [41]. In the current set of measurements, a colloidal probe indenter was driven into the PLL/HA film with a constant velocity, but unlike a standard elasticity measurement, the indenter was not retracted instantly after the initial indentation. It was left to dwell in the film while the *z*-piezo drive was paused. The AFM detector continued collecting the cantilever deflection data *F* as a function of time *t*. The dwell time was set to 40 s. A summary of this process is shown in Figure 8 and Figure 9: The colloidal probe indents into the film first due to the fast, short-time *z*-piezo driving (“Indentation” in Figure 9, mostly elastic response) and then due to a slow, long-time relaxation of the cantilever stress (“Relaxation” in Figure 9, viscous response). After 40 s (“Dwell Time” in Figure 9) the cantilever was retracted from the film, as in the case of a standard elasticity measurement (“Retraction” in Figure 9). Data points were collected every 0.1 s.

Typical *F* versus *t* relaxation curves on (PLL/HA)_72_ are presented in Figure 9 for two initial indentation velocities of 400 nm/s and 1000 nm/s. Regardless of the velocity, the cantilever stress relaxes totally to zero force. A total relaxation means that the film behaves as a viscoelastic liquid having either zero or very small equilibrium elasticity, in agreement with the results of reflection-interference-contrast microscopy from Picart and co-workers [23].

Figure 9 shows that for a fixed maximum load of 40 nN, the force decays slower for 400 nm/s than for 1000 nm/s. This behaviour was observed for other indentation velocities as well, indicating that the PLL/HA films act less viscously in the case of a faster initial indentation. In contrast to that, the instantaneous elastic response of the films is stronger for a faster indentation as shown in Figure 6.

For a quantitative comparison of the stress-relaxation behaviour for different initial indentation velocities, a multiexponential decay fit can be used as the model details can be found elsewhere [13–14]. Although not shown, an exponential decay with one relaxation component was unable to fit the curves. Therefore, the force relaxation was described with a biexponential decay with the assumption that the studied multilayer films have one short and one long relaxation scale, one corresponding to the dynamics of individual segments and one to the collective dynamics of many chains. For a spherical indenter of 3.35 μm in radius, two relaxation times *τ*_1_ and *τ*_2_ were calculated by fitting the *F* versus *t* curves by the biexponential decay below [13–14]:

[2]
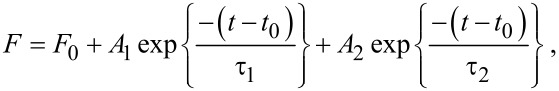


where *t*_0_ is the initial time and *F* is the force exerted on the cantilever at a time *t*. *F*_0_ corresponds to the elastic component of the relaxation. The fits are shown as thick lines in Figure 9. It should be noted that this fit is much simpler compared to the creep-compliance function [41,46], it resembles the stress relaxation fit used for heterogeneous materials [13–14], and allows for a qualitative comparison of the cantilever relaxation time rather than giving the actual material relaxation time.

Before discussing the outcome of the fits, two possible corrections should be mentioned. The first is the elimination of the effect of limited film thickness in the elastic component [13–14] *F*_0_, as discussed above. In an attempt, the effect of the limited film thickness was included in the fit function by using Equation 1; however, probably due to the small equilibrium value of the elastic component [23] *F*_0_, this correction did not result in a significant change in the fit, aside from making the fit function extremely complicated. The second possible correction can be performed to normalize the contact area of the colloidal probe during the stress relaxation, since the biexponential formula given above assumes a constant contact area during the process [13–14]. On the contrary, the contact area in our stress relaxation measurements changes as the colloidal probe moves deeper into the film. The change in the area can be calculated by using the (indentation-depth)–(time) relation, and the detected force at a certain time can be divided by the instantaneous contact area. Although the contact area may change by a maximum of 40% during the stress relaxation, this change results in an error in *τ*_1_ and *τ*_2_ that is much smaller than the uncertainty of the measurements, and thus the contact area was assumed to be constant for the sake of simplicity.

**Figure 10:**
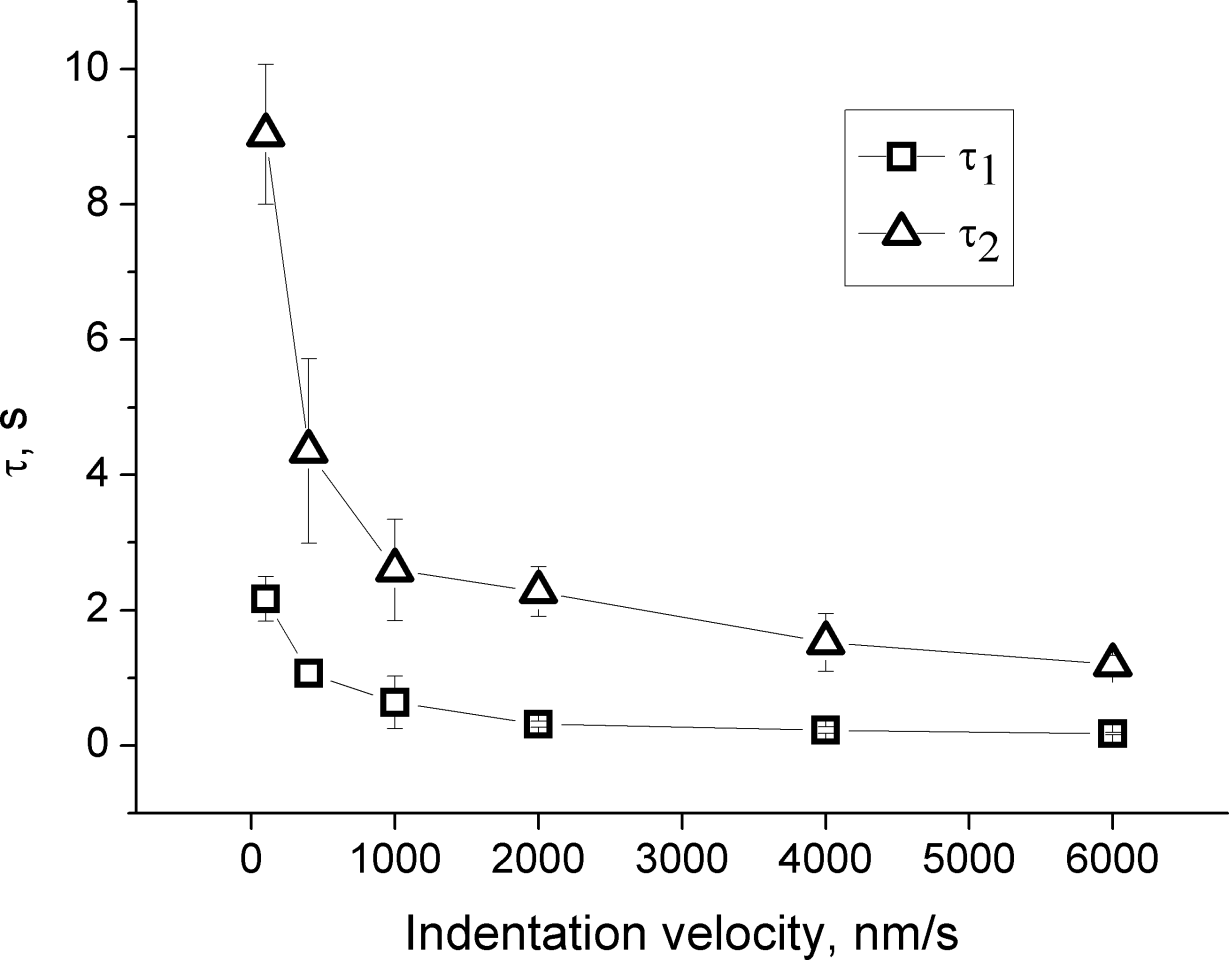
The cantilever’s stress relaxation time *τ*_1_ and *τ*_2_ as a function of the initial indentation velocity. Error bars indicate the standard deviation from 10 measurements.

The effect of the initial indentation velocity on *τ*_1_ and *τ*_2_ was studied with velocities of 100, 400, 1000, 2000, 4000 and 6000 nm/s at a maximum initial load of 40 nN. Ten measurements on different lateral positions were performed for each indentation velocity. The calculated *τ*_1_ and *τ*_2_ are presented in Figure 10.

Figure 10 shows a very clear dependence of *τ*_1_ and *τ*_2_ on the indentation velocity, indicating that PLL/HA films have a viscoelastic character, as has been previously suggested for similar multilayers [4–5,23,32], crosslinked PDMS films [47], human platelets [48], agar gel [49] and cancer cells [13–14]. From 100 nm/s to 6000 nm/s, *τ*_1_ continuously decreases from 2.17 s to 0.18 s and *τ*_2_ decreases from 9.03 s to 1.20 s. This decrease means that the film is less viscous when it is exposed to a faster initial load and gives a hint about a non-Newtonian, shear-thinning behaviour [50].

## Conclusion

Mechanical properties of layer-by-layer assembled PLL/HA films with varied bilayer number were studied by scanning- and colloidal-probe atomic force microscopy. Detailed measurement and data analysis techniques were addressed. Two independent AFM-based methods were used to measure the film thickness: Scratch-and-scan and full-indentation. Film thickness depends linearly on the bilayer number and ranges from ~400 nm to ~7500 nm for 12 and 96 bilayers, respectively. The apparent Young’s modulus of the films ranges between 15 to 40 kPa and the thinner films present larger error bars presumably due to the inhomogeneity of the surface. Film thickness and indenter size have no significant effect on the apparent Young’s modulus providing that the film-thickness-corrected Hertzian model is used to analyse the AFM force data. Multiple indentations at a fixed lateral film position can trigger a viscous or plastic deformation, continuously softening the structure.

Regardless of their thickness, PLL/HA films show a viscoelastic liquid behaviour. This is evidenced by the fact that the apparent Young’s modulus increases with indentation velocity and the cantilever stress relaxes to zero force after a while. Stress relaxation measurements show a biexponential decay indicating two relaxation processes, one due to the individual multilayer segments and the other to the collective film dynamics. Both relaxation times decrease with increasing initial indentation velocity, suggesting a non-Newtonian, shear-thinning fluid character. Frequency-dependent AFM force [41] and quartz crystal microbalance measurements are planned for a better understanding of shear and friction effects on the mechanical response of polymeric films.

## Experimental

### Preparation of polyelectrolyte films

The polyelectrolyte films PEI–(HA/PLL)*_n_*–HA, where *n* represents the number of deposited polymer pairs, were prepared by the layer-by-layer (LbL) technique [1] using a dipping robot (Riegler & Kirstein GmbH, Germany). The films were deposited on microscopy cover glasses (14 mm in diameter, Marienfeld GmbH, Germany). Before deposition, the glass slides were cleaned by consecutive incubation in hot solutions (60 °C) of 2% (w/v) Hellmanex (Hellma GmbH, Germany), 0.01 M sodium dodecyl sulphate, and 0.1 M HCl during 15 min for each solution followed by multiple rinsing with pure water. The film build-up was pursued at 25 °C by alternating dipping of the glass slides into PLL and HA solutions (0.5 mg/mL in 10 mM Tris-buffer containing 15 mM NaCl, pH 7.4) over 10 min with an intermediate washing step with the buffer (10 min). As with the precursor layer, PEI was adsorbed under the same conditions as other polymers. Before use, polyelectrolyte solutions were filtered through a 0.22 μm filter. The films were stored in Tris-buffer containing 0.15 M NaCl at 4 °C and never allowed to dry during the measurements.

### Microsphere attachment to the cantilever

Silica particles with a radius of 3.35 or 2.37 μm (Bangs Laboratories, Inc., USA) were used as the indenting probes. The probes were glued on CSC12 cantilevers (*μ*Masch, USA). Before the attachment process, the tipless cantilevers were cleaned in a plasma chamber for 20 min in order to get rid of any organic contaminants on their surface so that the glue spreads and adheres better. A two-component epoxy adhesive (UHU plus endfest 300, UHU GmbH, Bühl, Germany) was prepared and stored for 20 min under ambient conditions so that it is less fluid and easier to handle. The silica particles and the adhesive were placed on a glass slide. The adhesive was apportioned in fine stitches with a needle of a syringe or a very thin metal wire so that it had small separate droplets, ideally the size of the silica spheres. The cantilever was then moved by using a micro-manipulator. The far end of the cantilever was brought into contact with a drop of adhesive and finally was brought to a soft contact with an individual silica particle, leading to the attachment. After a successful attachment, the cantilever was stored for 24 h in ambient conditions. Finally, before each use, cantilevers were cleaned in an air plasma chamber for another 20 min.

### Cantilever calibration

Before each set of force measurements, the cantilever was calibrated in the medium (air, water, buffer, etc...) where the measurements were to be performed. This was done by bringing the cantilever into contact with a rigid surface and driving it further down by the piezo unit for a known distance. As there could not be any indentation on a hard surface, the driving distance was equal to the deflection of the cantilever. This step is crucial both for determination of the spring constant and for indentation measurements. Although the spring constant *k*_c_ was given by the manufacturer as 0.05 N/m (unless stated otherwise), its exact value was determined before each measurement by the thermal noise method, which is a built-in procedure in the MFP-3D instrument (Asylum Research, CA, USA).

### Thickness measurements

Two independent methods were performed with an MFP-3D AFM instrument (Asylum Research, CA, USA) in order to determine the thickness of the polyelectrolyte films. All measurements were performed as close to the centre of the substrate as possible to avoid any effect of film inhomogeneity.

#### Scratch-and-scan method

A part of the PEM film was removed by scratching it with a sharp needle or tweezers. Scratching the film in one direction with the correct angle led to a very small rim on one side of the scratch, as shown in Figure 1. The scratched area was imaged in the buffer medium with a magnetically driven *iDrive^™^* setup by using AR-iDrive-N01 (Asylum Research, USA) cantilevers. Scan rate was fixed to 0.1 Hz on a 90 μm × 90 μm area. The thickness of the film was calculated by using a cross-section profile on the AFM micrograph. Three different regions were scanned and up to five cross sections per image were used to obtain an average thickness. The three regions on the scratch were selected so that the distance between them was around 2 mm.

#### Full-indentation method

An AFM force measurement setup was used for indentation in the *z*-direction. The optical lever sensitivity was determined on a hard surface before any measurements on the soft films. *μ*Masch CSC37 cantilevers with a high spring constant *k*_c_ ≈ 0.3 N/m and a pyramidal tip with a length of 20–25 μm were used. The aim was not to measure any meaningful force but to penetrate through the film down to the hard substrate. When the tip reached the hard surface, the slope in the *F* versus *δ* curve changed drastically as indentation was no longer possible, as Figure 2 shows. The distance between the tip–film contact point and the tip–substrate contact point simply gave the thickness of the film. An average thickness was calculated over 10 to 20 measurements on different lateral positions.

### Elasticity measurements

Force measurements were performed with a commercial MFP-3D instrument (Asylum Research, CA, USA) by using the calibrated cantilevers with an attached silica microsphere. All measurements on (PLL/HA)*_n_* films were performed in a tris-buffer environment containing 0.1% NaN_3_ in order to prevent a bacterial contamination and a possible damage through drying. Force measurements were made with an indentation velocity of 50–6000 nm/s. The applied load on the films was controlled by a trigger point, which was set to a relative deflection corresponding to a 1.50 V detector signal after the contact. Unless stated otherwise, the lateral position of the measurement was changed after each indentation and the next measurement was taken on a spot around 50 to 100 μm away from the first one. Fifteen to thirty different spots were chosen in the central area of the substrate to calculate an average Young’s modulus *E*.

*E* was calculated from the raw *F* versus *δ* data by a thickness-corrected Hertzian model presented by Dimitriadis et al. [37]. The Young’s modulus of a film bonded to a hard substrate is given as

[1]
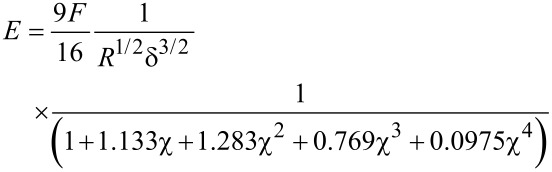


where *F* is the force exerted on the surface at an indentation depth *δ*, *R* is the radius of the spherical indenter and 
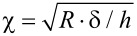
, *h* being the film thickness. The Poisson’s ratio was set to 0.5 for the PEM films due of their high water content, meaning that they are incompressible [37].

The above relation requires a precise determination of *h* and *R*. The thickness of the films *h* was determined by AFM as discussed above. The radius of the indenter curvature *R* was extracted from scanning electron microscope (SEM) images taken in TU Berlin ZELMI, with a high-resolution field emission microscope (S4000, Hitachi, acceleration field of 10 kV and 20 kV, no gold-coating).

Raw *F* versus *δ* data measured by CP-AFM were analysed by home-written procedures using Igor Pro software package (Wavemetrics Inc., USA). Average Young’s modulus was calculated in the indentation region 0.05*h* ≤ *δ* ≤ 0.2*h*. The probe–sample contact point was taken as the “jump-to-contact” of the probe to the surface. This point is shown in Figure 4a.

Viscoelasticity measurements were performed with some principle differences and the details are given in the Discussion section.
